# ADAM10 Is Involved in Cell Junction Assembly in Early Porcine Embryo Development

**DOI:** 10.1371/journal.pone.0152921

**Published:** 2016-04-04

**Authors:** Jeongwoo Kwon, Sung-min Jeong, Inchul Choi, Nam-Hyung Kim

**Affiliations:** 1 Department of Animal Sciences, Chungbuk National University, Naesudong-ro, Seowon-gu, Cheongju-si, Chungcheongbuk-do, 28864, Republic of Korea; 2 Department of Animal and Dairy Sciences, College of Agriculture and Life Sciences, Chungnam National University, Daejeon, 34134, Republic of Korea; Michigan State University, UNITED STATES

## Abstract

ADAM10 (A Disintegrin and Metalloprotease domain-containing protein 10) is a cell surface protein with a unique structure possessing both potential adhesion and protease domains. However, the role of ADAM10 in preimplantation stage embryos is not clear. In this study, we examined the expression patterns and functional roles of ADAM10 in porcine parthenotes during preimplantation development. The transcription level of ADAM10 dramatically increased from the morula stage onward. Immunostaining revealed that ADAM10 was present in both the nucleus and cytoplasm in early cleavage stage embryos, and localized to the apical region of the outer cells in morula and blastocyst embryos. Knockdown (KD) of *ADAM10* using double strand RNA did not alter preimplantation embryo development until morula stage, but resulted in significantly reduced development to blastocyst stage. Moreover, the KD blastocyst showed a decrease in gene expression of adherens and tight junction (AJ/TJ), and an increase in trophectoderm TJ permeability by disrupting TJ assembly. Treatment with an ADAM10 specific chemical inhibitor, GI254023X, at the morula stage also inhibited blastocyst development and led to disruption of TJ assembly. An *in situ* proximity ligation assay demonstrated direct interaction of ADAM10 with coxsackie virus and adenovirus receptor (CXADR), supporting the involvement of ADAM10 in TJ assembly. In conclusion, our findings strongly suggest that ADADM10 is important for blastocyst formation rather than compaction, particularly for TJ assembly and stabilization in preimplantation porcine parthenogenetic development.

## Introduction

Recently, we reported that CXADR (coxsackie virus and adenovirus receptor), a member of the junctional adhesion molecule (JAM) family of adhesion receptors, is necessary for the assembly of adherens junction (AJ) and tight junction (TJ) proteins during porcine preimplantation embryo development [1]. Interestingly, the CXADR knockdown (KD) study showed that CXADR plays critical roles in porcine embryo compaction and cavitation [1]. In line with previous studies [2], our recent finding provides clear evidence that CXADR is a component of TJ assembly and is essential for paracellular sealing on trophectoderm (TE) epithelial cells during blastocyst development [1]. In addition to its roles in AJ and TJ assembly, CXADR is also reported to be involved in physiological processes such as cell migration and regulation of growth [3, 4]. These events are mediated by ectodomain shedding and regulated intramembrane proteolysis (RIP); however, very little is known about the putative role of the shed (separated) ectodomain or cytoplasmic fragments of CXADR during preimplantation embryo development.

Here, we speculated that ADAM10 (A Disintegrin and Metalloprotease domain-containing protein 10) may be involved in the regulation of CXADR and in mediating CXADR RIP and ectodomain shedding. As a result, ADAM10 deficiency may also affect gene transcription in the absence of proteolytic cleavage of membrane proteins and fragments which act as transcription factors after translocation to the nucleus [5, 6].

There is a growing body of evidence supporting the interaction between CXADR and ADAM10 as essential for cell-cell adhesion and junction stability. A common AJ protein CDH1 (also known as E-cadherin), that is important for mammalian embryo compaction [7, 8] and required for the morphogenesis and maintenance of cells including cell growth and differentiation [9–11], can be cleaved specifically by ADAM10, consequently affecting cell adhesion, signaling, and apoptosis [12–15]. Furthermore, the phosphorylation of the C-terminus of CXADR is reported to play a key role in epithelial cell adhesion stability through control of CDH trafficking at cell-cell junctions [16]. ADAM10 belongs to the ADAMs family of transmembrane proteins that participate in several cellular process through multiple functional domains [17–19], including pro-, metalloproteinase, and disintegrin domains, as well as cysteine-rich, EGF-like, transmembrane, and cytoplasmic regions that contain SH3 domain binding motifs. Its extracellular domains have two catalytic domains, a peptidase and a disintegrin domain, that can cleave and bind various membrane proteins, including TNF-α and Notch, as well as various growth factors [20, 21]. The intracellular domain of ADAM10 can itself be shed, which can then translocate to the nucleus and regulate the expression of related genes [6]. During mouse embryogenesis, ADAM10 knockout experiments showed that it is a key protein in the central nervous system, somites, and cardiovascular system [22]. However, the biological roles of ADAM10 in terms of AJ and TJ assembly or biogenesis during early embryo development have not been elucidated.

In this study, we examined the expression patterns and localization of ADMA10 from one-cell embryo stage onward by qRT-PCR and immunocytochemistry (ICC), respectively. We employed a double-stranded RNA interference (dsRNAi) approach and treated embryos with a specific inhibitor of ADAM10, GI254023X, to investigate the biological functions of ADAM10 during preimplantation porcine embryo development. Our findings demonstrate that ADAM10 interacts with CXADR and is an essential molecule for TJ integrity and assembly in porcine embryo development.

## Materials and Methods

### Reagents

Unless otherwise noted, all chemicals were purchased from Sigma-Aldrich (St. Louis, MO, USA).

### *In vitro* porcine oocyte maturation (IVM)

All animal studies were performed in strict accordance with the institutional guideline and prior approval from the Institutional Animal Care and Use Committee (IACUC) of the Chungbuk National University.

Prepubertal porcine ovaries were transported from a local abattoir (Farm Story Hannang, Chungwon, Chungbuk, Republic of Korea) within one hour of harvest. Cumulus-oocyte complexes (COCs) were aspirated from 4–6 mm of ovarian follicles using 18-gauge needles attached to a 10-mL syringe. High-density cumulus oocytes were collected and washed three times with HEPE-PVA medium (HEPES medium supplemented with 0.01% polyvinyl alcohol). After washing, 60–80 COCs were cultured in 500 mL of IVM medium (M-199; Invitrogen, Carlsbad, CA) containing 20 ng/mL epidermal growth factor, 1 g/mL insulin, 75 g/ml kanamycin, 0.91 mM Na pyruvate, 0.57 mM l-cysteine, 10% (v/v) porcine follicular fluid, 0.5μg/mL follicle stimulating hormone (FSH), and 0.5μg/mL luteinizing hormone(LH) at 38.5°C in an atmosphere containing 5% CO_2_ at 100% humidity.

### Parthenogenetic activation and *in vitro* culture (IVC)

For parthenogenetic activation, mature oocytes were denuded by gentle pipetting in 1 mg/mL hyaluronidase until all the cumulus cells were removed from around the oocyte. Oocytes were then washed three times in PBS-BSA (DPBS added in 0.1% BSA), and activated using an Electro Cell Manipulator 2001 (BTX, Inc., San Diego, CA, USA). The electric pulse was stimulated at 1.1 kV/cm twice for 60 μsec in 280 mM mannitol medium supplemented with 0.01mM CaCl_2_ and 0.05mM MgCl_2_. Activated oocytes were treated in PZM-5 medium containing 7.5 μg/mL cytochalasin B in an incubator for 3 h at 38.5°C. Embryos were washed three times and cultured in PZM-5 for 144 h at 38.5°C in an atmosphere containing 5% CO_2_.

### Preparation of dsRNA and injections in porcine zygotes

ADAM10 dsRNA was designed using several experimental steps as previously described [23]. RNA was isolated using the Dynabeads mRNA Direct Kit (Life technologies AS, Oslo, Norway) from 50 porcine blastocysts, and cDNA was synthesized using the 1^st^ strand cDNA Synthesis Kit (Legene, San Diego, CA, USA) at 37°C for 45 min. ADAM10 was amplified using cDNA and an ADAM10 specific primer set listed in S1 Table. After electrophoresis and purification with a Gel Extraction Kit (Geneall biotechnology, Seoul, Republic of Korea), synthesized DNA was transcribed *in vitro* at 37°C for 4 h using the MEGAscript T7 Transcription Kit (Ambion, Austin, TX, USA). DNase I-treated RNA was purified by phenol-chloroform extraction.

After 8 h of electronic activation, ADAM10 dsRNA (1μg/μL) was microinjected into the zygote cytoplasm under a Nikon TE2000-U inverted microscope (Nikon Corporation; Tokyo, Japan) using an Eppendorf FemtoJet microinjector (Eppendorf; Hamburg, Germany). After injection, zygotes were cultured in PZM-5 medium for 144 h at 38.5°C in an atmosphere containing 5% CO_2_.

### Quantification of transcript levels

mRNA was isolated using the Dynabeads mRNA Direct Kit (Life technologies AS, Oslo) from one-cell, two-cell, four-cell, morula, and blastocyst stage embryos that were re-suspended in lysis/binding buffer and vortexed at room temperature, following adding 1ng GFP mRNA to the lysis/binding buffer (twenty embryos per a batch). cDNA synthesized with the 1^st^ strand cDNA Synthesis Kit (Legene, San Diego, CA, USA) was amplified using the DyNAmo SYBR Green qPCR Kit (Finnzymes Oy, Espoo, Finland) with gene specific primer pairs, listed in S1 Table, for relative quantification. The PCR cycling conditions were as follows: 95°C for 10 min, 39 cycles of 95°C for 10 sec, 55 or 60°C for 30 sec and 72°C for 30 sec, and finally extension at 72°C for 10 min. *GAPDH* was used as an internal control for normalization in all analyses.

### Immunofluorescence staining and confocal microscopy

To analyze immunofluorescence staining of cell junction proteins, embryos were fixed in 4% paraformaldehyde, followed by permeabilization in PBS containing 0.2% Triton X-100 for 1 h, and blocking in PBS containing 1% Bovine serum albumin and 0.05% Tween 20. The treated embryos were incubated overnight at 4°C with antibodies to either ADAM10 (sc-48400, Santa Cruz Biotechnology; Santa Cruz, CA, USA), CXADR (HPA003342, Sigma, St. Louis, MO, USA), TJP1 (ZO-1; 33–9100, Zymed, San Francisco, CA, USA), OCLN (Occludin; 33–1500, Zymed, San Francisco, CA, USA), CDH1 (E-cadherin; 610182, BD Biosciences, San Jose, CA), or CTNNB1 (β-catenin; sc-7963, Santa Cruz Biotechnology; Santa Cruz, CA, USA) that were diluted into blocking solution (1 in 100). After primary antibody incubation, the embryos were washed three times in PBS containing 0.05% Tween 20 for 10 min, and transferred to PBS containing Alexa-Fluor-488 diluted 1:200. Finally, the samples were stained with Hoechst 33342 (10 mg/mL in PVA-PBS) for 20 min. Before mounting, embryos were washed an additional three times, and mounted onto glass slides with Vectashield (94010, Vector Laboratories, Burlingame, CA). Each slide was examined using a confocal laser-scanning microscope (Zeiss LSM 710 META; Jena, Germany). At least 30 embryos were examined per group.

### Fluorescein isothiocyanate (FITC)-dextran uptake assay

The FITC-dextran uptake assay was carried out as previously described [1]. To examine TJ permeability, negative control and ADAM10 KD embryos were incubated in PZM-5 containing 1 mg/mL 40-kDa FITC-dextran for 30 min at 37°C. After incubation, the embryos were washed three times in PZM-5, transferred into fresh PZM5 covered with mineral oil, and analyzed under an inverted fluorescence microscope (Eclipse Ti-U, Tokyo, Japan)

### Treatment of embryos with GI254023X, a selective ADMA10 inhibitor

A specific inhibitor of ADAM10, GI254023X, is reported to inhibit the protein’s proteolytic activity and substrate shedding [24]. Porcine embryos were cultured in the presence or absence of 100 μM GI254023X from the morula stage (at 96 h.p.a) to the expanding blastocyst stage (at 144 h.p.a) for 48 h.

### Proximity ligation assay (PLA)

To detect protein–protein interactions between CXADR and ADAM10 in porcine blastocysts, a proximity ligation assay (PLA) was conducted using the *in situ* Red Starter Kit Mouse/Rabbit (DUO92101-1KT, Sigma, St. Louis, MO, USA). Embryos fixed in 3.7% formaldehyde were permeabilized with 0.2% Triton X-100 for 1 h, and then blocked in blocking buffer for 1 h. ADAM10 (anti-mouse) and CXADR (anti-rabbit) antibodies were diluted in blocking solution, and incubated with the embryos overnight at 4°C. After washing with PBS containing 0.05% Tween 20 three times, the embryos were incubated with the PLA PLUS and MINUS probes (a 1:5 dilution in antibody diluent solution) for 1 h at 37°C. The embryos were washed three times each for 5 min in washing buffer A (0.01 M Tris, 0.15 M NaCl, and 0.05% Tween 20 in high purity water). To ligate and circularize two DNA oligonucleotides, embryos were incubated in ligation-ligase solution (ligation mix 1:5, ligase 1:40, in high purity water) for 30 min at 37°C. After washing three times in washing buffer A, the embryos were transferred into the amplification and polymerase mixture (amplification mix 1:5, polymerase 1:8, in high purity water) for 100 min at 37°C, washed three times for 10 min in washing buffer B (0.2 M Tris and 0.1M NaCl in high purity water), and then mounted onto a slide with mounting medium containing DAPI. The PLA signals were visualized by a confocal laser-scanning microscope (Zeiss LSM 710 META; Jena, Germany).

### Data analysis

For each treatment, at least three biological and technical replications were performed. Analyses were carried out using the statistical software GraphPAD Prism program (GraphPad Software, La Jolla, CA). All data are presented as means ± standard error of the mean (s.e.m). The resulting data on embryo development and FITC dextran uptake assays were analyzed by the chi-square test, whiles differences in relative expression and diameter between the control and the KD or GI254023 treated embryos were calculated using one-way Analysis of Variance (ANOVA) followed by Tukey’s multiple comparison test. Statistical significance was determined at *P*<0.05.

## Results

### Expression patterns of ADAM10 in parthenogenetic preimplantation porcine embryos

**Fig 1:**
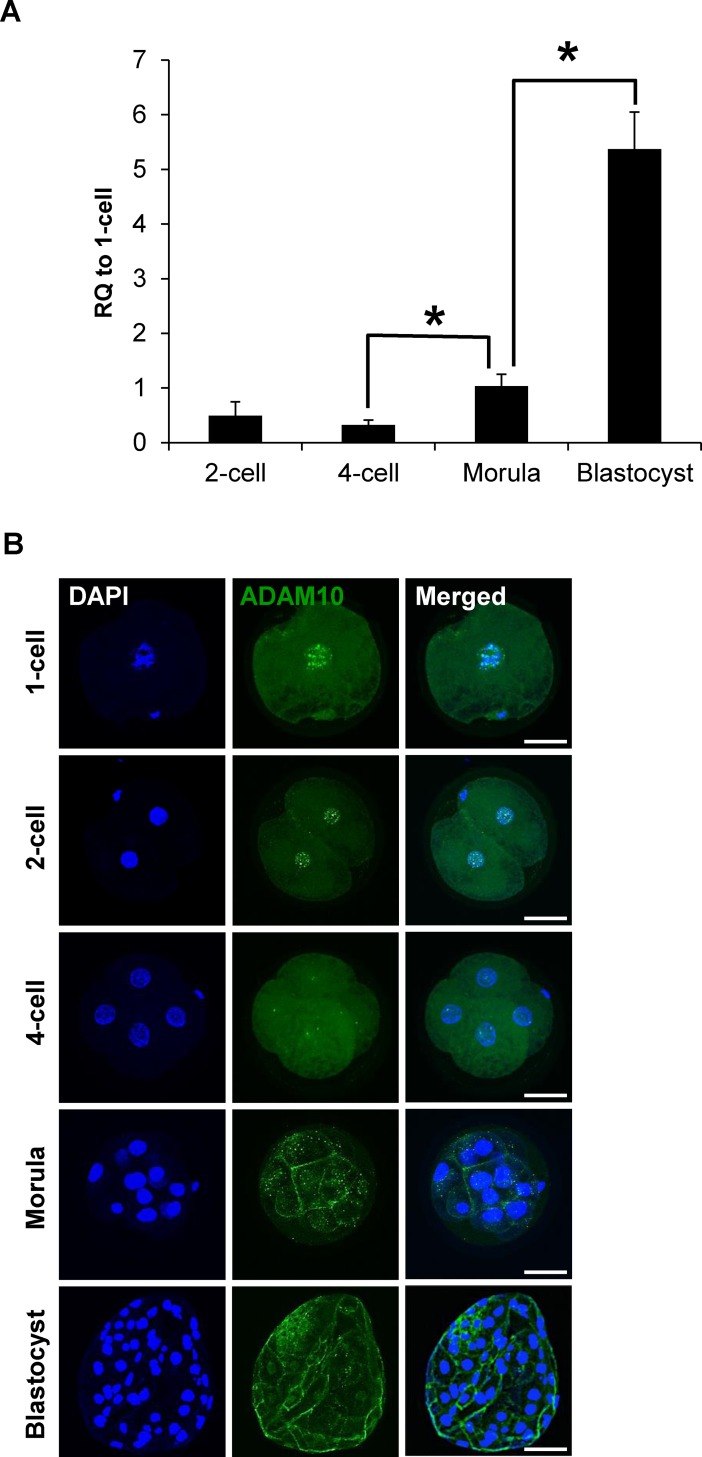
Expression patterns of ADAM10 during porcine embryo development. (A) Transcript levels of ADAM10 were upregulated from the morula stage and highest at the blastocyst stage; The quantitative RNA expression is presented as mean ± SEM; RQ: relative quantification. Asterisks indicate statistically significant differences (*P* < 0.05). (B) Analysis of expression and localization of ADAM10 in porcine embryos by immunocytochemistry (ICC). The ADAM10 signal was detected by an ADAM10 specific antibody (Green). Scale bars: 50 μm.

To determine the expression patterns of ADMA10 in porcine early embryos produced by parthenogenetic activation, we investigated the temporal changes in transcript levels of ADMA10 and its subcellular localization during porcine preimplantation development. ADAM10 mRNA levels were sharply increased from the morula stage onwards, and were significantly higher at the blastocyst stage (Fig 1A). Before embryo compaction (4 ~ 8 cells), ADAM10 was detected in both the nucleus and cytoplasm. Particularly, strong signals were observed in the nuclei of one- and two-cell embryos. At the morula stage, ADAM10 was mainly localized to sites of cell-to-cell contact. The localization of ADAM10 to the apical cell-cell boundaries became apparent in the blastocysts (Fig 1B). These results suggested that the translocalization and concentration of ADAM10 to the boundary of apical cell surfaces from pre-compaction to morula/blastocyst stages may be involved in AJ/TJ assembly.

### Effect of ADAM10 knockdown on embryo development

**Fig 2:**
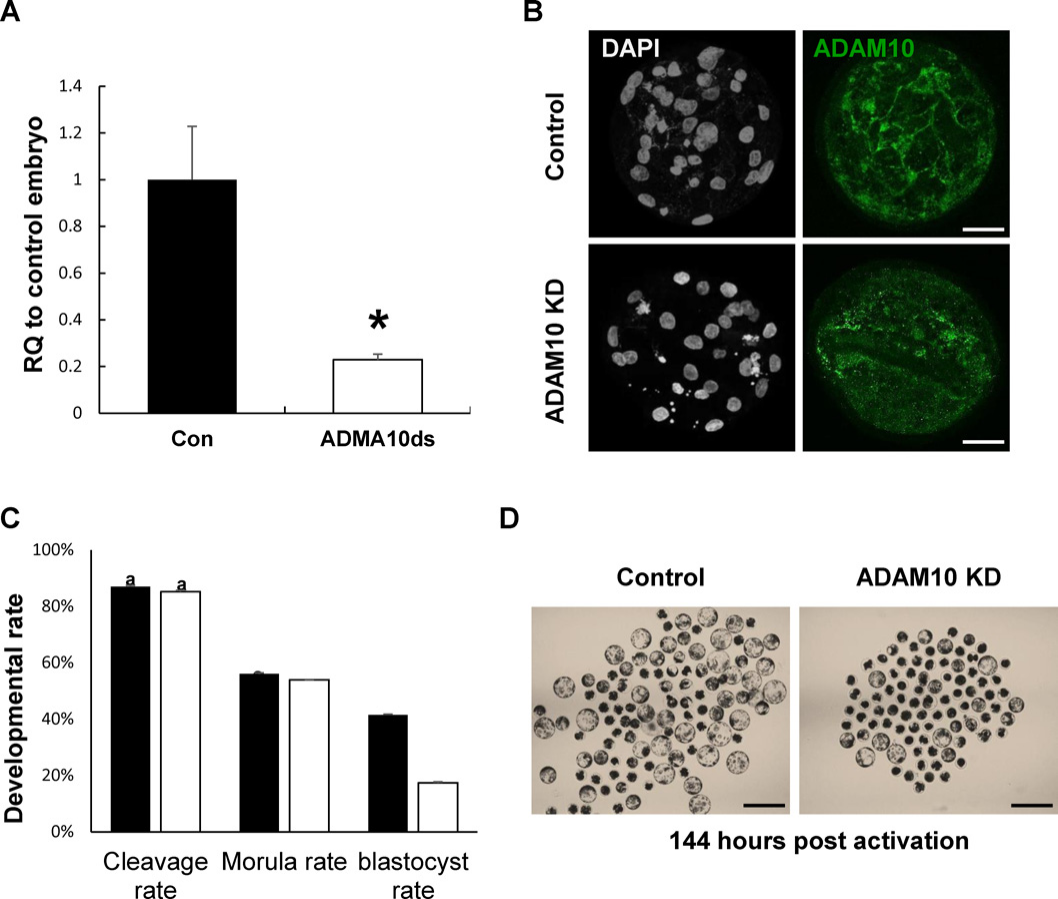
Knock-down (KD) of ADAM10 during early embryonic development. (A) KD efficiency of ADAM10 in blastocysts by qRT-PCR. (B) KD efficiency of ADAM10 in blastocysts by ICC. (C) Developmental competence of both control and knock-down groups. (D) Representative images of control and ADAM10 KD blastocysts at 144 h.p.a. Asterisks indicate statistically significant differences (*P* < 0.05). The data are presented as mean ± SEM. RQ: relative quantification; Scale bars: 200 μm.

To determine the functional roles of ADAM10 in the porcine early embryo development, we injected ADAM10 dsRNA into one-cell parthenotes and cultured them for 7 days in PZM-5. The efficiency of dsRNA mediated KD using qRT-PCR was 77.1% in the ADAM10 KD blastocysts (Fig 2A) and the protein was also depleted and not detected at the cell-cell contact boundaries, as seen Fig 2B. Interestingly, there was no difference in early developmental competence such as cleavage and morula development between the ADAM10 KD and control embryos, however, the blastocyst formation rate was significantly decreased in the KD groups (Fig 2C and 2D). These results suggested that ADAM10 may be involved in blastocyst formation rather than compaction.

### Disruption of TJ complex by ADAM10 KD

**Fig 3:**
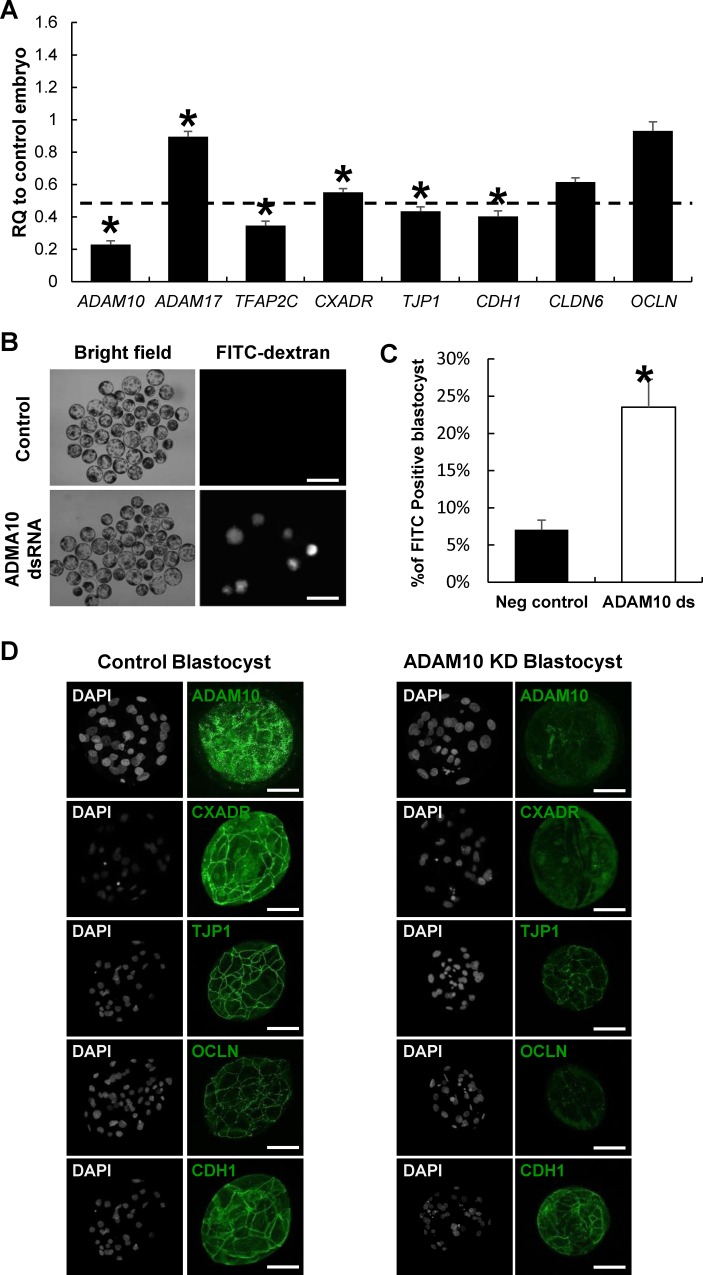
AJ and TJ disruption in ADAM10 KD embryos. (A) Altered expression of genes related AJ and TJ formation (*ADAM17*, *CXADR*, *TJP1*, *Cdh1*, *CLDN6*, *OCLN*, and *TFAP2C*). (B) FITC-Dextran uptake assay for paracellular permeability. ADAM10 KD blastocysts showed defects of paracellular sealing compared to the control. (C) Representative images of FTIC-dextran treated embryos under bright field and U.V. Scale bars: 200 μm. (D) AJ and TJ protein localization in ADMA10 KD embryos by ICC. In the KD blastocysts, TJ proteins (CXADR, TJP1, and OCLN) and the AJ protein (CDH1) were not localized to the apical region or reduced. Asterisks indicate statistically significant differences (*P* < 0.05). The data are presented as mean ± SEM. RQ: relative quantification. Scale bars: 50 μm.

Based on the reduced blastocyst formation in ADAM10 KD embryos, we speculated that ADAM10 adversely affects the expression of genes associated TJ complexes, particularly TJ assembly during the morula-to-blastocyst transition. To confirm the hypothesis, we examined transcript levels of AJ/TJ related genes at the blastocyst stage. In the ADAM10 KD blastocysts, the transcription levels of AJ/TJ related genes including CXADR, OCLN, TJP1, CDH1, and CLDN6 were significantly reduced (Fig 3A). To analyze whether KD of ADAM10 affects paracellular permeability, we employed FITC-dextran uptake assay that is usually used to examine the barrier function of the cell-to cell boundaries of blastocysts [25], incubated both control and ADAM10 KD blastocysts with 40 kDa FITC-Dextran, and counted the FITC-positive signals under UV light. The FITC-dextran accumulated in 23.53% of the ADAM10 KD blastocyst cavities, but it was detected in only 7.05% of that of the control blastocysts (Fig 3B and 3C). In line with the reduced transcription levels of AJ/TJ genes in the ADAM10 KD, the result of the FITC uptake assay indicated a functional defect in paracellular sealing, which is established by proper TJ biogenesis/assembly, in the KD blastocyst. Thus, we evaluated AJ and TJ proteins in the ADAM10 KD blastocyst, and found that TJ transmembrane proteins, including CXADR and OCLN, were barely detectible, particularly at the apical region of the cell-cell boundaries, and adaptor protein TJP1 was localized to the apical region but the protein amount was reduced (Fig 3D). Furthermore, CDH1 was not present in clear continuous lines at cell-to-cell boundaries, compared with that of the control. The results of the gene and protein expression analyses in the ADAM10 KD blastocysts support the notion that ADAM10 is more important for tight junction assembly than adhesion junction formation.

### Effect of the treatment of embryos with an ADAM10-specific inhibitor on blastocyst formation

**Fig 4:**
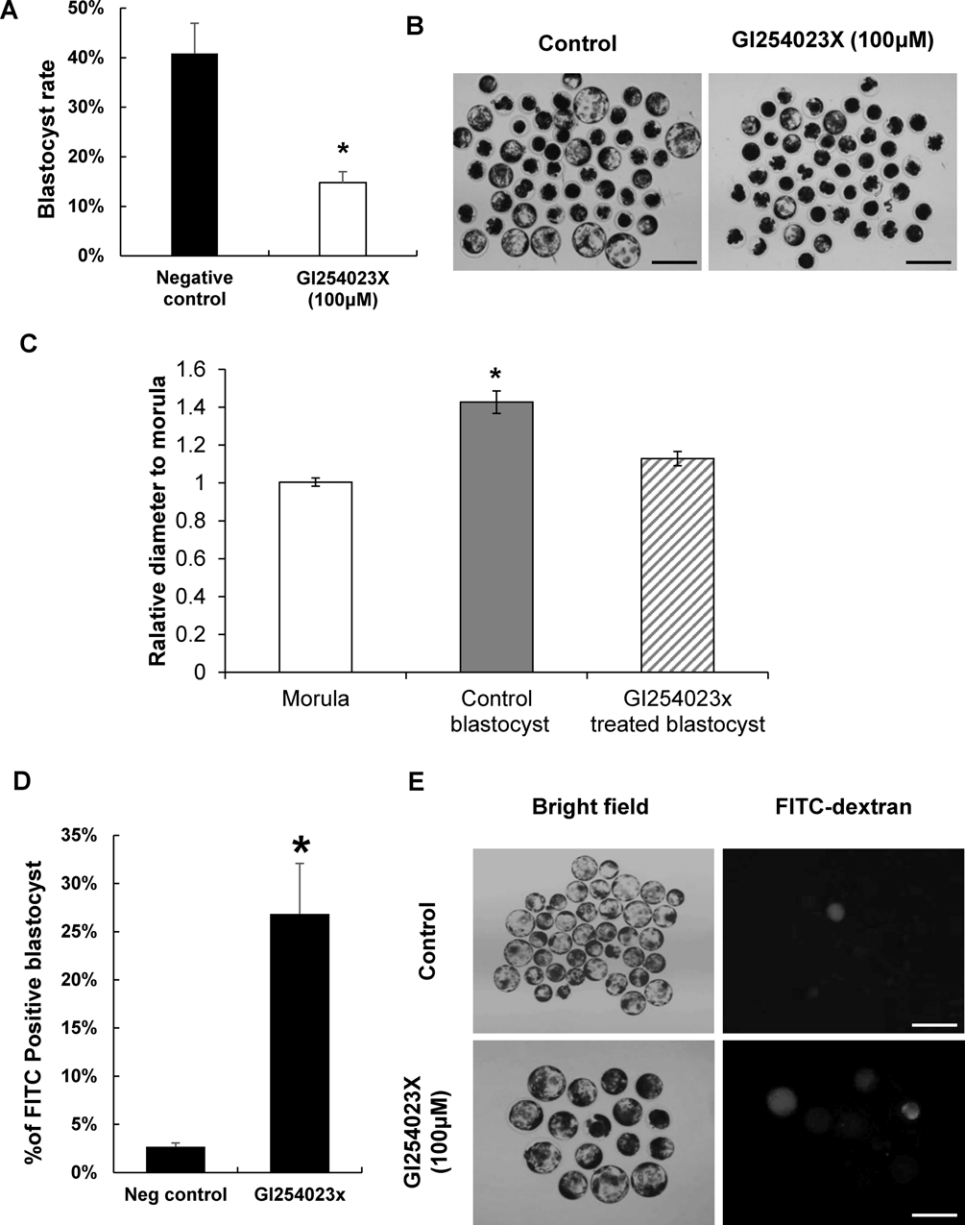
Treatment of morula embryos with ADAM10 inhibitor GI254023X. (A) Effects of the ADAM10 specific inhibitor on the transition from morula to blastocyst stage. (B) Representative images of control and GI254023X treated embryos from 96 h to 144 h. (C) Blastocyst embryos from the morula, control blastocyst, and GI254023x treated blastocyst were collected, and their diameters were measured; the diameters were compared with those in the morula (morula: empty bars). The ratio denotes the relative expansion of the control and GI254023x treated blastocysts (control blastocyst: filled bars; GI254023x treated blastocyst: hatched bars). (D) FITC-Dextran uptake assay for paracellular permeability. GI254023x treated blastocyst showed defects of paracellular sealing, compared to the control. Asterisks indicate statistically significant differences (*P* < 0.05). (E) Representative images of FTIC-dextran treated embryos under bright field and U.V. Scale bars: 200 μm.

To understand the inhibitory effects of ADAM10 activity on blastocyst formation, including cavitation and TJ integrity during the morula-blastocyst transition in porcine embryos, we exposed morula staged embryos to the ADAM10 specific inhibitor GI254023X for 48 h and compared their development with non-treated controls. We found that blastocyst formation rates were significantly lower in the ADAM10 inhibitor treated group than those in the non-treated controls (14.77%. vs. 40.86%; Fig 4A and 4B). Particularly, the ADAM10 inhibitor treated embryos were relatively not expanded but control morula embryos were able to cavitate and expand (relative ratio of diameter to morula, 1.12±0.03 vs. 1.42±0.05, P<0.05; Fig 4C), indicating disruption of TJ assembly/biogenesis during the transition stage. The FITC-Dextran uptake assay supported that inhibition of ADAM10 activity led to a defect of TJ assembly (Fig 4D).

### Interaction of ADAM10 with junctional adhesion molecule CXADR in the trophectodermal cells of blastocysts

**Fig 5:**
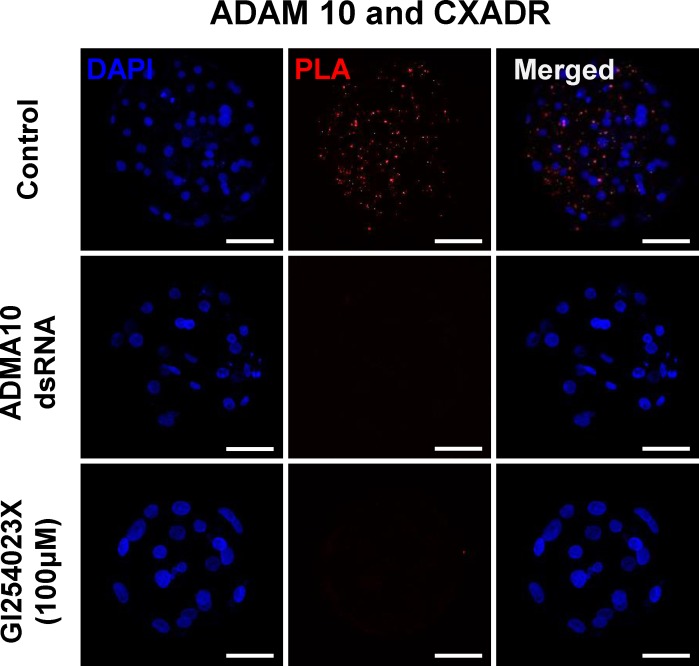
Proximity ligation assay (PLA) in blastocysts and hypothetical model for the roles of ADAM10 in embryo development. ADAM10 and CXADR interaction in porcine blastocyst using PLA. Control: 144 h.p.a in PZM-5 (non-treatment); ADAM10 dsRNA: ADAM10 dsRNA injection after 8 h.p.a and culturing for 136 h; GI254023X (100 μM): GI254023X treatment after 96 h.p.a to 144 h.p.a.

We carried out an *in situ* proximity ligation assay (PLA) in order to determine whether ADAM10 and CXADR form a complex necessary for the assembly of tight junction proteins including OCLN and TJP1. Red dots were detected in the control blastocysts, but no PLA signal was observed in the ADAM10 KD and activity inhibited blastocysts (Fig 5). As expected, the red dots were observed exclusively in the cytoplasm, and appeared to be localized to cell-cell contact sites.

## Discussion

Previous studies showed that CXADR is a component of TJs and may interact with TJ proteins to maintain their integrity [1, 26]. Recently, some studies have suggested that CXADR and CDH1 are associated with the stability of cell-cell junctions, and epithelial cell–cell adhesion is mediated by ADAM10 [14, 16, 27]. However, several studies have reported that ADAM10 mediated cleavage of adhesion proteins, including CDH1, may contribute to junction disassembly, and inhibition of the proteolytic activity, or ADAM10 KD prevents endothelial barrier disruption [28–31].

To elucidate the functional roles of ADAM10 and its putative interaction with CXADR in porcine parthenotes during early preimplantation, we first examined the expression patterns of the parthenotes and performed a loss-of-function study using RNAi. In this study, we found that ADAM10 was upregulated and became localized to cell-cell boundaries from the morula stage onwards. This localization may reflect its involvement in AJ/TJ assembly and disassembly. Interestingly, we found no significant differences in cleavage and morula development between the ADAM10 KD and control embryos. However, the majority of ADAM10 KD morula embryos failed to form blastocysts, suggesting that ADAM10 is involved in TJ assembly rather than AJ assembly or junction disassembly. In addition, the findings of down-regulation of genes associated with AJ and TJ complexes in the ADAM10 KD blastocysts, except for OCLN suggested that depletion of ADAM10 affects different types of signaling and transcriptional pathways linked to TJ associated gene expression [32] although the underlying mechanism behind the altered gene expression in the KD embryos is not yet clear. Furthermore, the reduction and mislocalization of TJ proteins of the KD blastocysts in ICC assay, and the remarkable increase in paracellular permeability in the TE epithelium of ADAM10 KD blastocyst in the FITC dextran uptake assay support the involvement of ADAM10 in TJ assembly/integrity.

To provide further evidence to support the results of ADAM10 KD studies, we suppressed proteolytic activity of ADAM10. The treatment of morula embryos with the ADAM10 specific inhibitor GI254023X adversely affected blastocyst formation including blastocyst cavity expansion and paracellular sealing. These findings reinforce our assumption that ADAM10 plays a critical role in the clustering of TJ proteins during morula and blastocyst transition, and the expansion of blastocysts mediated by the stabilization of TJ integrity.

**Fig 6:**
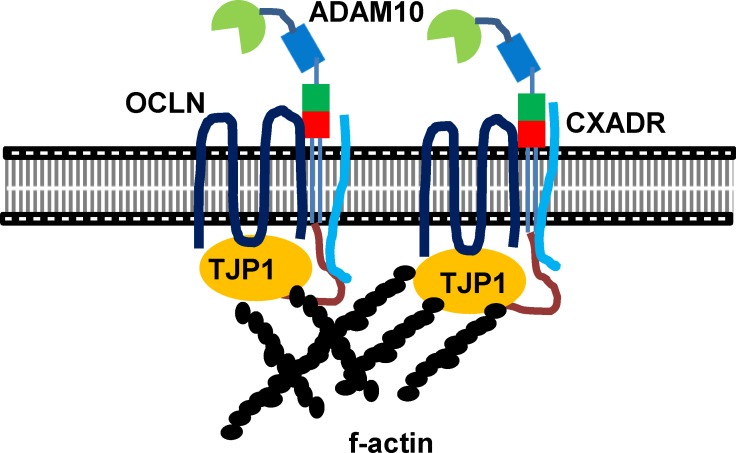
A model of ADAM10 during blastocyst formation. The SH3 domains of CXADR and TJP1 interact with SH2 binding motif of the cytoplasmic domain of ADAM10. Tetraspanin proteins such as OCLN interact with the extracellular domains of ADAM10 or with TJP1, suggesting that the clustering of AJ and TJ mediated by ADAM10 provides strong TJ assembly and paracellular sealing, and leads to cavitation and expansion from the transition stage between morula and blastocyst and onwards.

Particularly, applying the in situ PLA to porcine embryos demonstrated that ADAM10 interacts with a TJ molecular constituents, CXADR that is important for TJ assembly in embryo development [1, 26]. Surprisingly, we found no interaction of ADAM10 with CXADR in the GI254023X treated blastocysts. Therefore, we question how closely CXADR and ADAM10 are clustered or interact, and why the inhibition of ADAM10 proteolytic activity affects their interaction. Given the patterns of ADAM10 expression and its direct interaction with TJ proteins including CXADR, we postulate that the intracellular regions of ADAM10 serve as docking sites for SH3 domains of single-pass (CXADR) and four-pass (OCLN, CLDNs) membrane proteins at cell-cell contact points, a process mediated by the SH3 domains of adapter proteins such as TJP1 [2, 33–37], and that ADAM10 may also interact with the large extracellular loop of four-pass membrane proteins, supporting stabilization of TJ assembly [38] (Fig 6). Interestingly, overall C-shaped ADAM structure seems to be crucial for the interaction between extracellular loop and cysteine rich region of ADAM 10. In this context, treatment of GI254023X modifies proteolytic aspect of ADAM10 and conformational changes of extracellular domain of ADAM 10, leading to prevention of the interaction [19, 39–41].

Our data reveal that expression patterns and localization of ADAM 10 during parthenogenetic porcine preimplantation development, and the interaction of ADAM10 and CXADR to form TJ complex in the morula to blastocyst transition

We conclude that ADAM10 is required for sequential TJ assembly of constituents such as CXADR, TJP1, and OCLN for blastocyst development, and TJ integrity mediated by ADAM10 on TE blastocyst is directly involved in expansion of the blastocyst. This is a novel finding that ADAM10 is a component of TJ complex as well as a sheddase.

## Supporting Information

S1 Table Primer sequences for dsRNA and qRT-PCR. dsRNA: ADAM10 dsRNA for knock-down primer. qRT-PCR: mRNA expression check primer.
(DOCX)
