# Supplementary material for: ADAM10 Is Involved in Cell Junction Assembly in Early Porcine Embryo Development
Source: PLoS One. 2016 Apr 4;11(4):e0152921. doi: 10.1371/journal.pone.0152921 (PMC4820119; doi:10.1371/journal.pone.0152921)
Supplement: S1 Table — dsRNA: ADAM10 dsRNA for knock-down primer. qRT-PCR: mRNA expression check primer. (DOCX) [file pone.0152921.s001.docx]

**Supplementary table 1.** **Primer sequences for dsRNA and qRT-PCR.**

| **Gene** | **GenBank accession No.** | | **Sequence (5’– 3’)** | **Amplicon size (bp)** |
| --- | --- | --- | --- | --- |
| ***ADAM10 dsRNA*** | AF109646.1 | F: TAATACGACTCACTATAGCGAGACCACGCAACATCTGGGGACAAACT  R: TAATACGACTCACTATAGCGAGACCACATGGTTCGACCAAGGAAGTG | | 611 |
| ***ADAM10*** | NM_001130531 | F: GGCTTGGAGGAGTGTACCTG  R: GCTAGAGGACCGTCAGCATC | | 239 |
| ***ADAM17*** | NM_001099926.1 | F: CAGGCCTCAACACACCTAGA  R: CACTTGACGGGGTACTCACT | | 201 |
| ***TFAP2c*** | NM_001123201 | F: GAAACCCTGGACTGGACGAG  R: GTAGCACCACTTGCAGAGGA | | 121 |
| ***CLDN6*** | XM_005662194 | F: CCTGTAAGACGGAGCCAGAG  R: GTGTCCAGGGTGGAGAAAGA | | 64 |
| ***CXADR*** | AF109646.1 | F: GAAACCCTGGACTGGACGAG  R: GTAGCACCACTTGCAGAGGA | | 121 |
| ***OCLN*** | NM_001163647 | F: GCRGGAGGAAGACTGGAT  R: ATCCGCAGATCCCTTAAC | | 244 |
| ***TJP1*** | CK453343 | F: ACCCACCAAACCCACCAA  R: CCATCTCTTGCTGCCAAACTATC | | 123 |
| ***CDH1*** | NM_001163060 | F: ACTGGGTTATCCCTCCCATC  R: AAACGGGCCTTTCTCATTTT | | 50 |
| ***eGFP*** | YP_009062989.1 | F: GCAAGCTGACCCTGAAGTTCA  R: TCACCTTGATGCCGTTCTTCT | | 796 |

**(**F: forward. R: reverse)

s
